# Association of Social Media‐Driven Cosmetic Consumption With Skin Barrier Damage, Delayed Medical Consultation, and Disease Severity in Acne Vulgaris: A Physician‐Assessed Cross‐Sectional Study

**DOI:** 10.1111/jocd.71020

**Published:** 2026-07-02

**Authors:** Orhan Şen, Atıl Avcı

**Affiliations:** ^1^ Department of Dermatology Kayseri City Hospital Kayseri Turkey; ^2^ Department of Dermatology Kayseri University Faculty of Medicine Kayseri Turkey

**Keywords:** acne vulgaris, barrier damage, cosmetic polypharmacy, health literacy, patient behavior, social media

## Abstract

**Objective:**

Social media content influences cosmetic consumption among acne patients; however, its clinical consequences remain incompletely understood. This study aimed to evaluate the association of social media‐driven cosmetic consumption, information sources, and cosmetic awareness levels with clinical outcomes (barrier damage, acne severity, delayed medical consultation) in acne vulgaris patients.

**Methods:**

A cross‐sectional, descriptive study was conducted in 408 acne patients presenting to a dermatology outpatient clinic. Social media usage habits, dermocosmetic term awareness, and product consumption behaviors were assessed using a structured data collection form, while acne severity, barrier damage, and cosmetic polypharmacy history were evaluated by an attending dermatologist. Data were analyzed using chi‐square test, Mann–Whitney *U* test, Spearman correlation, and multivariate logistic regression.

**Results:**

The mean age of participants was 20.9 ± 5.1 years, and 77.5% were female. Barrier damage was significantly higher in patients who purchased products through social media (64.3% vs. 10.2%; *p* < 0.001). In multivariate analysis, social media‐driven product purchase (OR = 3.25; *p* = 0.002) and delayed medical consultation (OR = 6.65; *p* < 0.001) were identified as independent risk factors for barrier damage. TikTok users exhibited greater delays in seeking medical care (66.7% vs. 49.0%; *p* = 0.028) and higher rates of severe acne compared with Instagram users. Paradoxically, a higher number of known dermocosmetic terms was associated with increased barrier damage risk and delayed consultation, a pattern that may reflect superficial rather than clinically applicable knowledge. Conversely, following dermatologist accounts emerged as an independent protective factor against barrier damage (OR = 0.28; *p* = 0.001).

**Conclusion:**

Social media‐driven cosmetic consumption is strongly associated with barrier damage and worsening clinical disease severity in acne patients. Increasing evidence‐based content production by dermatologists on social media platforms may positively contribute to the patient care process.

## 1 Introduction

Acne vulgaris is a chronic inflammatory disease of the pilosebaceous unit and one of the most prevalent dermatological conditions, particularly affecting the adolescent and young adult population [1, 2].

It is well established that acne vulgaris is not merely a physical disease but also adversely affects the psychosocial well‐being, self‐esteem, and quality of life of affected individuals. The onset of acne vulgaris during adolescence and young adulthood coincides with a period of heightened sensitivity to physical appearance and intensified desire for social acceptance. This predisposes patients to seeking rapid and accessible solutions; however, with the digitalization of information access channels, this pursuit has increasingly shifted away from medical consultation toward social media platforms [3, 4].

Social media platforms have become one of the most widely used channels for accessing health information. These rapidly expanding platforms have emerged as prominent tools for information dissemination and health information seeking across multiple domains, including healthcare [5].

Although dermatologic content production on social media platforms has increased rapidly in recent years, a substantial proportion of this content is generated by individuals without formal medical training [6, 7]. Acne vulgaris is among the most frequently shared dermatologic topics on social media [8], reaching large audiences through skincare recommendations, product promotions, and personal experience narratives. However, significant concerns exist regarding the scientific accuracy and reliability of this content. Studies have demonstrated that the majority of dermatologic content on social media is not evidence‐based, and that content produced by non‐expert sources receives substantially greater reach and engagement compared with expert‐generated content. This increases the risk of patients being exposed to misinformation and using unverified products [9].

The existing literature has largely documented the impact of social media on dermatology patients' information‐seeking behaviors and treatment preferences through patient‐reported survey studies. However, the number of studies that directly correlate social media‐driven cosmetic consumption behavior with clinician‐verified clinical outcomes (skin barrier damage, acne severity, cosmetic usage history) remains very limited. Most of these studies rely solely on patient self‐report and lack clinical verification [10, 11, 12, 13].

Furthermore, the differential impact of social media platforms (TikTok, Instagram, YouTube) on clinical outcomes has not been sufficiently investigated. Additionally, the relationship between patients' cosmetic literacy and their actual product use behaviors has not yet been examined. The number of studies that comparatively evaluate how information obtained from different sources translates into clinical‐level outcomes remains quite limited in the existing literature [11, 12].

This study aims to determine the association between social media‐driven cosmetic consumption behavior and clinician‐verified clinical outcomes in acne vulgaris patients. The effects of social media‐driven product purchasing, platform preference, type of followed accounts, and cosmetic literacy level on barrier damage, acne severity, and timing of medical consultation were evaluated using multivariate analyzes.

## 2 Materials and Methods

### 2.1 Study Design, Setting, and Sample

This cross‐sectional, descriptive study was prospectively conducted among patients presenting with acne vulgaris complaints to a dermatology outpatient clinic.

### 2.2 Inclusion and Exclusion Criteria

Inclusion criteria were defined as follows: being aged 12–45 years, receiving a clinical diagnosis of acne vulgaris by the attending dermatologist, and having sufficient cognitive ability to independently complete the data collection form. Exclusion criteria were defined as follows: systemic medication use that could cause acneiform eruptions (corticosteroids, lithium, antiepileptics, etc.), presence of a concurrent inflammatory facial dermatosis (rosacea, perioral dermatitis, seborrheic dermatitis), and refusal to participate in the study or provide consent. In accordance with these criteria, a total of 408 patients who met the eligibility requirements during the study period were enrolled using consecutive sampling.

### 2.3 Ethical Approval

Ethical approval for this study was obtained from the institutional ethics committee. The study was conducted in accordance with the principles of the Declaration of Helsinki and the Good Clinical Practice Guidelines. Written informed consent was obtained from all participants, and from the legal guardians of patients under 18 years of age.

### 2.4 Data Collection Instrument

Data were collected using a structured data collection form developed by the researchers based on the current literature, incorporating both patient‐reported and physician‐assessed components. The form consisted of four sections, with the first three completed by the patient and the fourth completed by the physician based on clinical examination findings.

#### 2.4.1 Section 1—Demographics

Age, gender, education level (primary, secondary, high school, university, postgraduate), duration of acne complaints (0–6 months, 6–12 months, 1–3 years, > 3 years), and history of previous medical consultation for acne treatment were recorded.

#### 2.4.2 Section 2—Social Media Usage Habits

Daily social media usage duration (none, < 1 h, 1–3 h, > 3 h), the most frequently used platform for skincare information (Instagram, TikTok, YouTube, Google/websites, none), and types of social media accounts followed (dermatologists, influencers/content creators, beauty professionals/aestheticians, celebrities; multiple selections allowed) were assessed. Patients who reported not using social media at all (*n* = 12) were categorized under “Not using social media for skincare information” for the information source variable. The “Physician only” category was reserved for patients who used social media but reported obtaining skincare information exclusively from their physician.

#### 2.4.3 Section 3—Cosmetic Literacy and Product Use

Whether patients had purchased skincare products influenced by social media, the number of cosmetic products regularly used on the face (none, 1–2 types, 3–5 types, > 5), whether skin deterioration occurred after using a product seen on social media, and whether social media recommendations delayed medical consultation were assessed. Dermocosmetic term awareness was also evaluated.

#### 2.4.4 Section 4—Physician Assessment

This section was completed exclusively by the attending dermatologist based on clinical examination findings. Acne severity, presence of barrier damage, and cosmetic usage history were evaluated.

### 2.5 Dermocosmetic Term Awareness

As no standardized cosmetic literacy scale for acne patients exists in the literature, seven key terms most frequently encountered in current social media content and the dermocosmetic literature were identified by the researchers to assess patients' familiarity with dermocosmetic concepts: retinol, salicylic acid, vitamin C, niacinamide, alpha hydroxy acid/beta hydroxy acid (AHA/BHA), skin barrier, and double cleansing. Patients were individually asked whether they had heard of or knew each term, and responses were recorded as aware (1) or unaware (0) for each term. It should be emphasized that this 7‐item structure was not designed as a psychometrically validated scale of cosmetic literacy; rather, it served as a pragmatic indicator of patients' exposure to commonly circulated dermocosmetic terminology. Findings related to term awareness should therefore be interpreted within this methodological constraint.

### 2.6 Clinical Assessment Parameters

#### 2.6.1 Acne Severity

Acne severity was assessed by the attending dermatologist during routine clinical examination based on the Investigator's Global Assessment (IGA) classification and categorized into three clinical categories: mild (comedonal and/or mild papulopustular lesions), moderate (widespread papulopustular lesions), and severe (nodulocystic/conglobate lesions).

#### 2.6.2 Barrier Damage

The presence of skin barrier damage was assessed by the attending dermatologist during routine clinical examination using predefined, standardized clinical criteria. Barrier damage was recorded as present when at least two of the following three findings co‐existed on the facial skin: (i) visible erythema beyond the active acne lesions (diffuse or perilesional), (ii) fine desquamation or scaling indicating compromised stratum corneum integrity, and (iii) patient‐reported subjective symptoms of burning, stinging, or tightness consistent with an irritant reaction. To enhance internal consistency, all clinical assessments were performed by the same attending dermatologist throughout the study period, thereby eliminating inter‐observer variability. The diagnosis of irritant contact dermatitis associated with cosmetic product use was made in line with widely accepted clinical descriptions in the dermatologic literature. Objective biophysical methods such as transepidermal water loss (TEWL) measurement, corneometry, or skin pH‐metry were not available within the routine outpatient setting and were therefore not used in this study; this limitation is explicitly addressed in Section 4.1.

#### 2.6.3 Cosmetic Usage History

The patient's cosmetic product usage profile was classified by the physician into three categories: polypharmacy (concurrent use of multiple dermocosmetic products beyond the basic skincare routine recommended in current guidelines for acne patients—cleanser, active treatment agent, moisturizer, and sunscreen—without clinical indication), insufficient (lacking a basic cleansing and moisturizing routine), and appropriate (balanced cosmetic use consistent with the clinical condition) [14, 15].

### 2.7 Statistical Analysis

The sample size was calculated as a minimum of 88 patients for the chi‐square test based on an α = 0.05 significance level, 80% statistical power, and a medium effect size assumption (Cohen's w = 0.30), and a minimum of 393 patients for multivariate logistic regression analysis according to the Events Per Variable ≥ 10 rule. A total of 408 patients were enrolled during the study period, exceeding these requirements.

Categorical variables were presented as numbers (*n*) and percentages (%), while continuous variables were presented as mean ± standard deviation (SD) and median (min–max) values. Associations between categorical variables were assessed using Pearson's chi‐square test. The Mann–Whitney *U* test was used for two‐group comparisons and the Kruskal‐Wallis test for comparisons involving three or more groups of non‐normally distributed continuous variables. Correlations between ordinal variables were examined using Spearman's rank correlation analysis. To evaluate the relationship between cosmetic awareness level and clinical outcomes, the number of terms known by each patient (0–7) was calculated as an analytical variable and entered into multivariate logistic regression models as a continuous variable; the reported odds ratio therefore represents the change in odds per one additional term known.

To identify independent predictors of barrier damage, delayed medical consultation, severe acne, and social media‐driven product purchasing, separate multivariate logistic regression models were constructed for each dependent variable. Candidate variables were selected through a two‐step process: (i) clinical and theoretical relevance based on the existing literature on social media use and dermatologic outcomes, and (ii) statistical screening through univariate analyzes, with variables showing *p* < 0.25 considered eligible for inclusion, in accordance with the recommendations of Hosmer and Lemeshow. Potential risk factors meeting these criteria (age, gender, daily social media usage duration, social media‐driven product purchasing, influencer following, dermatologist following, number of products used, number of known terms, delayed medical consultation, information source preference) were entered into the models using the Enter method rather than stepwise selection, in order to retain theoretically relevant variables and to avoid the instability and overfitting often associated with data‐driven selection procedures in moderate‐sized datasets. Prior to model construction, multicollinearity among predictor variables was assessed using Variance Inflation Factor (VIF) and tolerance values. All VIF values were below 2.0 (maximum VIF = 1.81) and tolerance values exceeded 0.55, indicating the absence of problematic multicollinearity among the predictors entered into the final models. Pairwise Spearman correlations between social media–related variables (social media‐driven purchase, influencer following, dermatologist following, SM duration, and number of known terms) ranged from 0.05 to 0.50 in absolute value, supporting the conclusion that these variables capture related but distinct constructs. To address potential confounding by clinically relevant covariates not initially included in the primary models, a pre‐specified sensitivity analysis was additionally performed for the barrier damage outcome, in which prior acne treatment history and education level (used as a proxy for socioeconomic status) were entered into Model 1 as additional covariates, and the stability of the principal effect estimates was evaluated. Model fit was assessed using the Hosmer‐Lemeshow test, and explanatory power was evaluated using the McFadden Pseudo *R* ^2^ value. Results were presented as odds ratios (OR) with 95% confidence intervals (95% CI). All statistical analyzes were performed using IBM SPSS Statistics version 28.0 (IBM Corp., Armonk, NY, USA). A *p*‐value of < 0.05 was considered statistically significant for all tests.

## 3 Results

### 3.1 Demographic and Clinical Characteristics

A total of 408 patients were enrolled in the study. The mean age was 20.9 ± 5.1 years (range: 12–45), and 77.5% (*n* = 316) were female. Regarding education level, 48.3% had a high school education and 46.3% had a university or higher education level. Patients with acne duration exceeding 1 year constituted 68.4% of the sample, and 66.9% had previously consulted a physician for acne treatment. Clinical assessment revealed mild acne in 28.4%, moderate acne in 46.8%, and severe acne in 24.8% of patients. Barrier damage was detected in 25.5%. In the cosmetic usage history assessment, 17.2% were classified as polypharmacy, 43.4% as insufficient, and 39.5% as appropriate. Demographic and clinical characteristics are summarized in Table 1. Gender‐based comparison revealed that the rate of social media‐driven product purchasing was significantly higher in females than in males (32.0% vs. 15.2%; *p* = 0.003), while no statistically significant difference was found for barrier damage (27.5% vs. 18.5%; *p* = 0.106) or delayed consultation (36.7% vs. 25.0%; *p* = 0.050). When evaluated by age groups, social media‐driven product purchasing and delayed consultation rates were highest in the 18–25 age group (33.9% and 40.3%, respectively). The association between social media‐driven product purchasing and barrier damage was significant across all three age groups (12–17, 18–25, and 26–44 years) (all *p* < 0.001).

**TABLE 1:** Demographic and clinical characteristics of the study population (*n* = 408).

Characteristic	*n* or mean	(%) or ± SD
Age (years), mean ± SD	20.9 ± 5.1	
Gender, *n* (%)		
Female	316	(77.5)
Male	92	(22.5)
Education, *n* (%)		
Primary/Secondary	22	(5.4)
High school	197	(48.3)
University/Postgraduate	189	(46.3)
Duration of acne, *n* (%)		
0–6 months	72	(17.6)
6–12 months	57	(14.0)
1–3 years	137	(33.6)
> 3 years	142	(34.8)
Previous treatment, *n* (%)	273	(66.9)
Acne severity (IGA), *n* (%)		
Mild	116	(28.4)
Moderate	191	(46.8)
Severe	101	(24.8)
Barrier damage, *n* (%)	104	(25.5)
Cosmetic history, *n* (%)		
Polypharmacy	70	(17.2)
Insufficient	177	(43.4)
Appropriate	161	(39.5)
Daily SM usage, *n* (%)		
None	12	(2.9)
< 1 h	33	(8.1)
1–3 h	198	(48.5)
> 3 h	165	(40.4)
SM‐driven product purchase, *n* (%)	115	(28.2)
Delayed medical consultation, *n* (%)	139	(34.1)
Information source, *n* (%)		
Not using SM for skin info	129	(31.6)
Instagram	104	(25.5)
TikTok	75	(18.4)
YouTube	22	(5.4)
Google/Websites	41	(10.0)
Physician only	37	(9.1)
SM accounts followed,^a^ *n* (%)		
Dermatologists	181	(44.4)
Influencers	128	(31.4)
Beauty professionals	73	(17.9)
Celebrities	27	(6.6)
Terms known, mean ± SD	3.6 ± 2.3	

Abbreviations: IGA, Investigator's Global Assessment; SM, social media.

^a^ Multiple selections allowed.

### 3.2 Social Media Usage Findings

Of all patients, 97.1% (*n* = 396) used social media, with 48.5% using it 1–3 h daily and 40.4% using it more than 3 h daily. The most frequently preferred platforms for skincare information were Instagram (25.5%), TikTok (18.4%), and Google (10.0%), while 31.6% reported not using social media for skincare information. Regarding the types of social media accounts followed, 44.4% followed dermatologists, 31.4% followed influencers, 17.9% followed beauty professionals, and 6.6% followed celebrities.

Among all patients, 28.2% (*n* = 115) reported purchasing a product they had seen on social media, and 34.1% (*n* = 139) reported delaying medical consultation due to social media recommendations. Assessment of dermocosmetic term awareness revealed that the most commonly known terms were vitamin C (82.8%), salicylic acid (60.5%), and double cleansing (53.9%), while the least known terms were AHA/BHA (30.6%) and niacinamide (35.3%). The mean number of known terms was 3.6 ± 2.3.

### 3.3 Association Between Social Media Behaviors and Clinical Outcomes

The rate of barrier damage was 64.3% in patients who purchased products through social media compared with 10.2% in non‐purchasers (χ^2^ = 124.5; *p* < 0.001; OR = 15.82; 95% CI: 9.25–27.07). Similarly, the rate of delayed medical consultation was 76.5% in product purchasers versus 17.4% in non‐purchasers (OR = 15.47; 95% CI: 9.14–26.18). The rate of severe acne was 41.7% in the purchasing group versus 18.1% in the non‐purchasing group (*p* < 0.001).

Among patients who followed influencers, the rates of barrier damage, delayed consultation, and social media‐driven product purchasing were 53.1%, 68.0%, and 61.7%, respectively, compared with 12.9%, 18.6%, and 12.9% in non‐followers (all comparisons *p* < 0.001). A significant dose–response relationship was observed between the number of products used and barrier damage: barrier damage rates were 3.9% in non‐users, 20.4% in those using 1–2 products, 64.0% in those using 3–5 products, and 77.8% in those using more than 5 products (Spearman rho = 0.463; *p* < 0.001) (Table 2).

**TABLE 2:** Univariate associations between social media behaviors and barrier damage.

Variable	Barrier (+) *n* = 104	Barrier (−) *n* = 304	*p*	OR (95% CI)
SM‐driven purchase				
Yes	74 (64.3%)	41 (35.7%)	< 0.001	15.82 (9.25–27.07)
No	30 (10.2%)	263 (89.8%)		Ref.
Influencer follow				
Yes	68 (53.1%)	60 (46.9%)	< 0.001	7.68 (4.69–12.58)
No	36 (12.9%)	244 (87.1%)		Ref.
Delayed consultation				
Yes	84 (60.4%)	55 (39.6%)	< 0.001	19.01 (10.77–33.57)
No	20 (7.4%)	249 (92.6%)		Ref.
Product count				
None	4 (3.9%)	99 (96.1%)	< 0.001^a^	—
1–2	45 (20.4%)	176 (79.6%)		—
3–5	48 (64.0%)	27 (36.0%)		—
> 5	7 (77.8%)	2 (22.2%)		—
Information source				
Not using SM	8 (6.2%)	121 (93.8%)	< 0.001	—
Instagram	41 (39.4%)	63 (60.6%)		—
TikTok	33 (44.0%)	42 (56.0%)		—
YouTube	5 (22.7%)	17 (77.3%)		—
Google	9 (22.0%)	32 (78.0%)		—
Physician only	8 (21.6%)	29 (78.4%)		—
Cosmetic history				
Polypharmacy	61 (87.1%)	9 (12.9%)	< 0.001	—
Insufficient	40 (22.6%)	137 (77.4%)		—
Appropriate	3 (1.9%)	158 (98.1%)		—

Abbreviations: CI, confidence interval; OR, odds ratio; Ref., reference category.

^a^ Spearman rank correlation test. Cross‐sectional design precludes causal inference; associations are presented for descriptive purposes.

### 3.4 Platform Comparison

**FIGURE 1:**
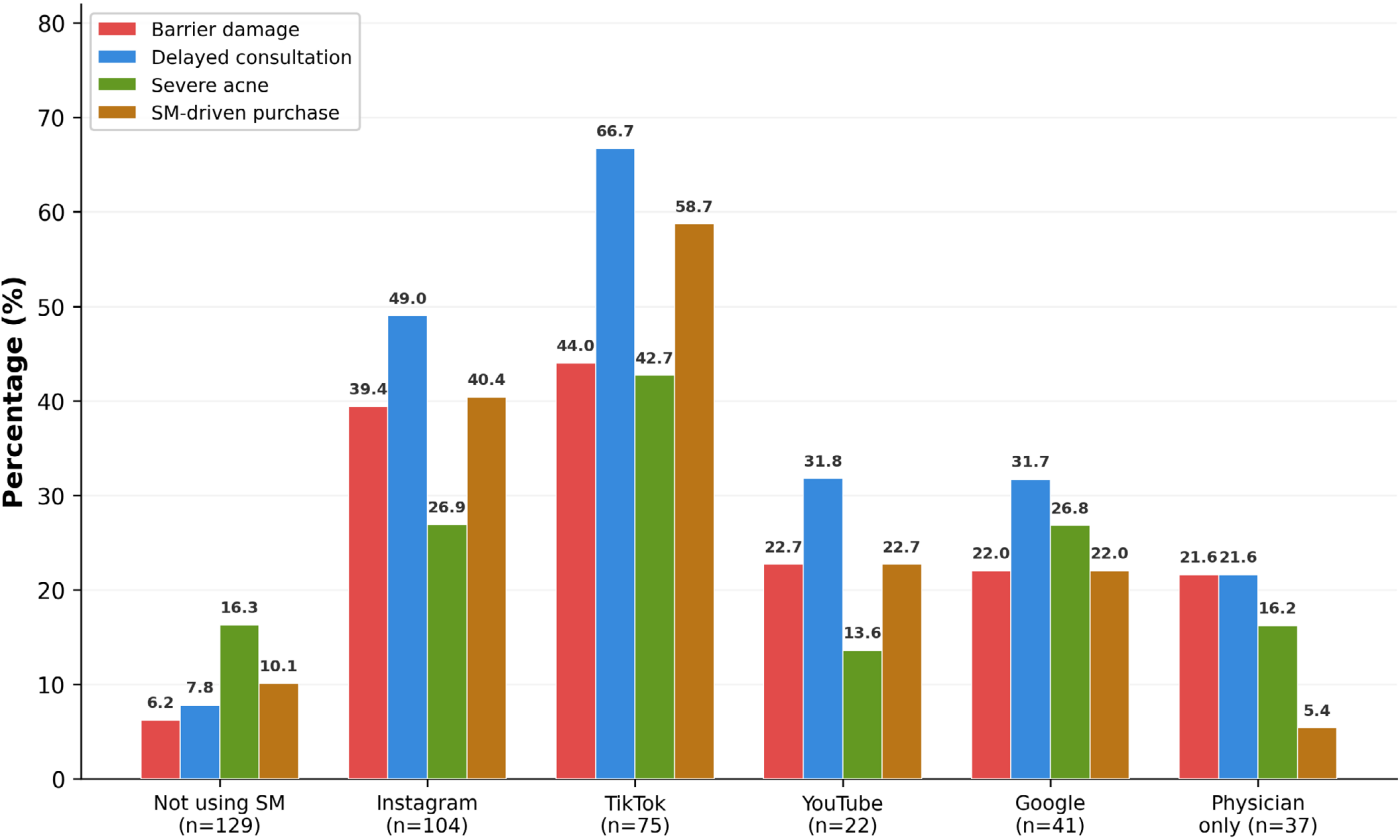
Comparison of social media‐driven product purchasing, delayed medical consultation, and acne severity between TikTok and Instagram users.

When clinical outcomes were compared among patients using different platforms as information sources, TikTok users demonstrated the highest rates of social media‐driven product purchasing (58.7%), delayed medical consultation (66.7%), and severe acne (42.7%). In direct comparison with Instagram users, the rate of social media‐driven product purchasing was significantly higher among TikTok users (58.7% vs. 40.4%; *p* = 0.024). Delayed medical consultation was also significantly more prevalent in the TikTok group (66.7% vs. 49.0%; *p* = 0.028). In terms of acne severity, TikTok users had a significantly higher mean severity score compared with Instagram users (2.31 vs. 2.03; *p* = 0.010) (Figure 1).

### 3.5 Comparison of Dermatologist and Influencer Following

**FIGURE 2:**
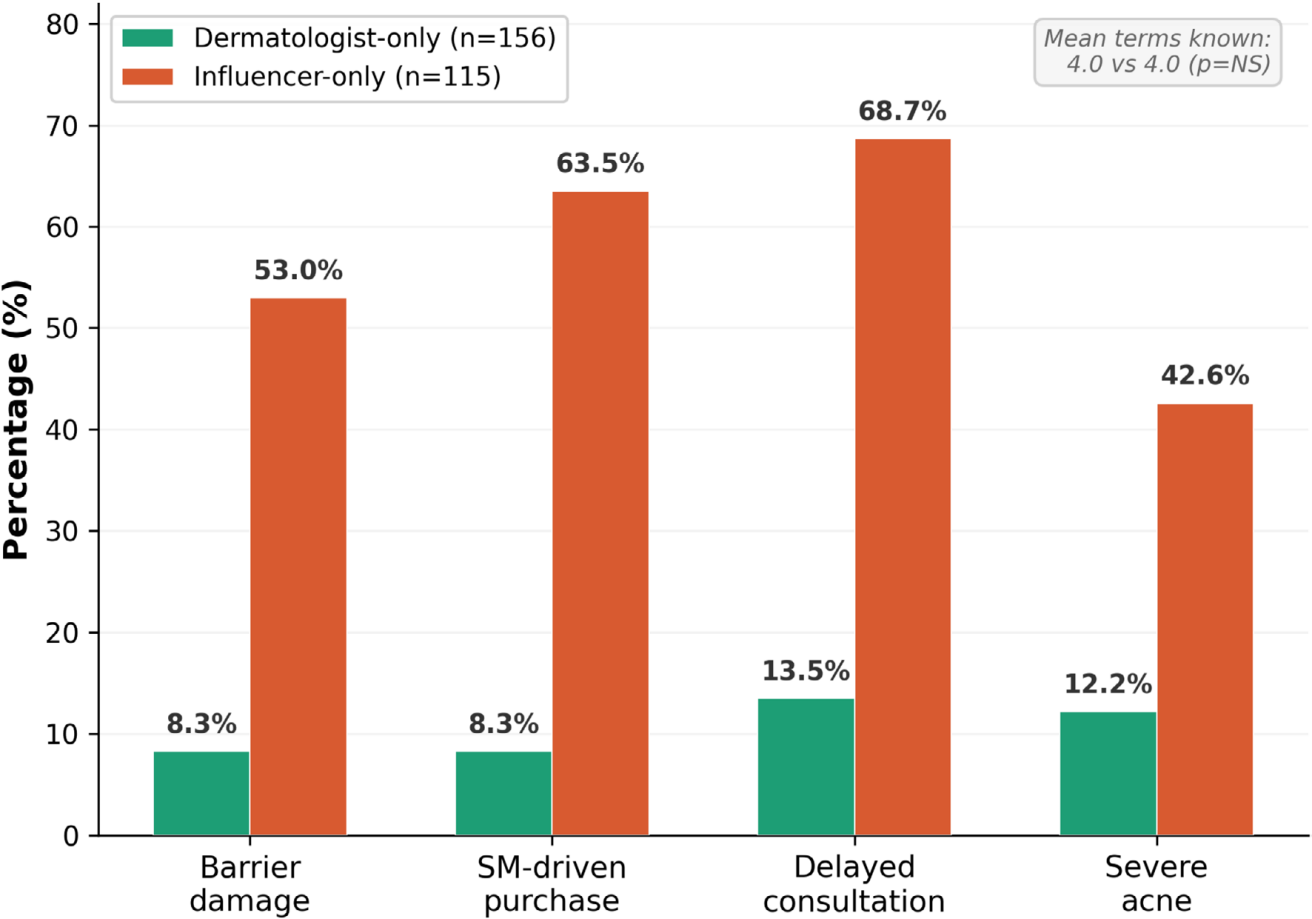
Comparison of clinical outcomes between patients following only dermatologist accounts and those following only influencer accounts.

When patients who followed only dermatologists (*n* = 156) were compared with those who followed only influencers (*n* = 115), notable differences in clinical outcomes were observed despite equivalent mean numbers of known terms (4.0 vs. 4.0). The barrier damage rate was 8.3% in dermatologist‐only followers versus 53.0% in influencer‐only followers. The rates of social media‐driven product purchasing (8.3% vs. 63.5%), delayed medical consultation (13.5% vs. 68.7%), and severe acne (12.2% vs. 42.6%) were similarly and significantly higher in the influencer‐following group (all comparisons *p* < 0.001; chi‐square test) (Figure 2).

### 3.6 Cosmetic Awareness Level and Clinical Outcomes

**FIGURE 3:**
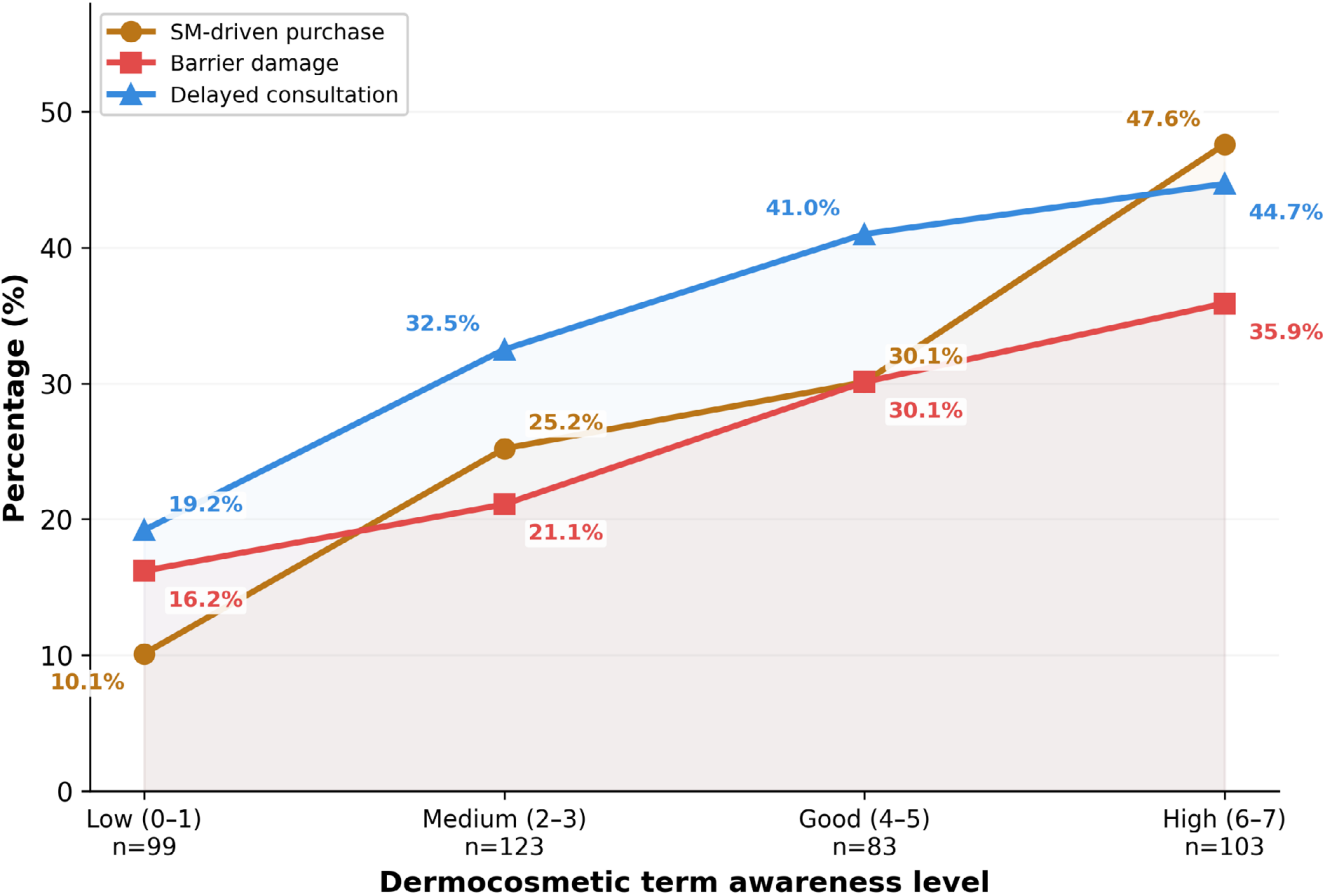
Association between the number of known dermocosmetic terms and rates of social media‐driven product purchasing, barrier damage, and delayed medical consultation.

When patients were categorized into four groups based on the number of known terms, increasing term awareness was associated with rising rates of social media‐driven product purchasing, barrier damage, and delayed consultation. In the low‐awareness group (0–1 terms, *n* = 99), social media‐driven purchasing was 10.1%, barrier damage was 16.2%, and delayed consultation was 19.2%, whereas in the high‐awareness group (6–7 terms, *n* = 103), these rates were 47.6%, 35.9%, and 44.7%, respectively (all comparisons *p* < 0.05) (Figure 3).

### 3.7 Multivariate Logistic Regression Analysis

Preliminary diagnostics confirmed no problematic multicollinearity among predictor variables (maximum VIF = 1.81; minimum tolerance = 0.55), and Hosmer‐Lemeshow goodness‐of‐fit *p*‐values were > 0.05 for all four models, indicating adequate fit. In the multivariate model constructed for barrier damage (Pseudo *R* ^2^ = 0.435), delayed medical consultation (OR = 6.65; 95% CI: 3.38–13.08; *p* < 0.001), number of products used (OR = 3.94; 95% CI: 2.21–7.01; *p* < 0.001), and social media‐driven product purchasing (OR = 3.25; 95% CI: 1.57–6.72; *p* = 0.002) were identified as independent risk factors. Age, gender, social media usage duration, and number of known terms were not significant as independent risk factors. In the pre‐specified sensitivity analysis incorporating prior acne treatment history and education level as additional covariates, the principal associations remained essentially unchanged: delayed medical consultation (OR = 5.98; 95% CI: 3.00–11.94; *p* < 0.001), number of products used (OR = 3.91; 95% CI: 2.23–6.85; *p* < 0.001), and social media‐driven product purchase (OR = 3.21; 95% CI: 1.59–6.49; *p* = 0.001) all retained their independent associations with barrier damage. Neither prior treatment history (OR = 0.64; 95% CI: 0.32–1.26; *p* = 0.196) nor education level (OR = 1.15; 95% CI: 0.63–2.09; *p* = 0.645) was independently associated with barrier damage, and the model's explanatory power changed only marginally (Pseudo *R* ^2^ from 0.435 to 0.438). All VIF values remained below 2.0 in the sensitivity model, supporting the robustness of the primary findings.

In the model constructed for delayed medical consultation (Pseudo *R* ^2^ = 0.310), social media‐driven product purchasing (OR = 8.34; *p* < 0.001) and influencer following (OR = 4.04; *p* < 0.001) were identified as independent predictors. The severe acne model (Pseudo *R* ^2^ = 0.138) demonstrated that delayed consultation (OR = 3.03; *p* < 0.001), barrier damage (OR = 2.54; *p* = 0.006), and male gender (OR = 2.20; *p* = 0.014) were independent risk factors.

In the model constructed for social media‐driven product purchasing behavior (Pseudo *R* ^2^ = 0.370), influencer following (OR = 5.68; *p* < 0.001), celebrity following (OR = 5.81; *p* = 0.001), beauty professional following (OR = 3.15; *p* = 0.001), and the number of known terms (OR = 1.55; *p* < 0.001) increased purchasing behavior, while dermatologist following was identified as a protective factor (OR = 0.28; 95% CI: 0.13–0.60; *p* = 0.001) (Table 3).

**TABLE 3:** Multivariate logistic regression models for clinical outcomes and social media behaviors.

Independent variable	OR	95% CI	*p*
Model 1: Barrier Damage (Pseudo *R* ^2^ = 0.435)			
Delayed consultation	6.65	3.38–13.08	< 0.001
Product count	3.94	2.21–7.01	< 0.001
SM‐driven purchase	3.25	1.57–6.72	0.002
Influencer follow	1.83	0.86–3.88	0.115
SM duration	1.29	0.80–2.09	0.300
Age	0.98	0.91–1.05	0.551
Gender (male)	1.53	0.64–3.68	0.338
Model 2: Delayed Consultation (Pseudo *R* ^2^ = 0.310)			
SM‐driven purchase	8.34	4.49–15.48	< 0.001
Influencer follow	4.04	2.16–7.54	< 0.001
Information source	1.22	1.04–1.43	0.016
SM duration	1.03	0.70–1.51	0.877
Gender (male)	0.92	0.45–1.87	0.808
Dermatologist follow	0.83	0.44–1.60	0.585
Model 3: Severe Acne (Pseudo *R* ^2^ = 0.138)			
Delayed consultation	3.03	1.62–5.64	< 0.001
Barrier damage	2.54	1.31–4.95	0.006
Gender (male)	2.20	1.18–4.10	0.014
SM‐driven purchase	1.47	0.76–2.83	0.253
Acne duration	1.20	0.95–1.53	0.132
Model 4: SM‐Driven Purchase (Pseudo *R* ^2^ = 0.370)			
Influencer follow	5.68	2.98–10.83	< 0.001
Celebrity follow	5.81	2.03–16.61	0.001
Beauty prof. follow	3.15	1.57–6.34	0.001
Terms known (0–7)	1.55	1.32–1.82	< 0.001
SM duration	1.43	0.93–2.22	0.105
Dermatologist follow	0.28	0.13–0.60	0.001

*Note:* Only variables with *p* < 0.25 in univariate analysis were entered into models using Enter method. Non‐significant variables were retained to adjust for potential confounding. Model fit assessed by Hosmer‐Lemeshow (H‐L) test: Model 1 H‐L *p* = 0.089; Model 2 H‐L *p* = 0.121; Model 3 H‐L *p* = 0.271; Model 4 H‐L *p* = 0.126 (all *p* > 0.05, indicating adequate fit). McFadden Pseudo *R* ^2^ reported for each model. Multicollinearity diagnostics: all VIF values < 2.0 (maximum 1.81), all tolerance values > 0.55.

Abbreviations: CI, confidence interval; OR, odds ratio.

## 4 Discussion

In this physician‐assessed cross‐sectional study, social media‐driven cosmetic consumption among acne vulgaris patients was examined in relation to clinician‐verified outcomes, namely skin barrier damage, acne severity, and the timing of medical consultation, using multivariate analyses.

In our study, the barrier damage rate was 64.3% in patients who purchased products through social media versus 10.2% in non‐purchasers, and this association was confirmed as an independent risk factor in multivariate analysis (OR = 3.25; *p* = 0.002). These findings strengthen the limited clinical data in the existing literature demonstrating that social media‐driven cosmetic use is associated with dermatologic harm. A recently published systematic review reported that skincare trends promoted on social media are mostly not evidence‐based and have a heterogeneous impact on users' skin health behaviors [16]. Our study distinguishes itself from previous studies by demonstrating this effect not only at the patient self‐report level but at the clinical level through physician assessment.

It is well known that products containing active ingredients (acids, retinoids, vitamin C) are widely recommended on social media platforms. In a recently published editorial, it was emphasized that the use of multiple inappropriate products driven by social media trends increases the risk of irritant contact dermatitis, skin barrier disruption, and allergic sensitization, particularly in young patients [17, 18]. Our data numerically support this clinical observation: a significant dose–response relationship was identified between the number of products used and barrier damage, consistent with a dose–response pattern, although causality cannot be inferred from the cross‐sectional design.

One of the most notable findings of our study is that 34.1% of patients delayed medical consultation due to social media recommendations, and this delay was identified as the strongest independent risk factor for both barrier damage (OR = 6.65) and severe acne (OR = 3.03). Ertekin et al. in their multicenter study from Turkey, reported that approximately 21% of adult female acne patients were willing to alter their physician‐prescribed treatment based on social media recommendations [11]. Our study demonstrates that this tendency manifests not only as treatment modification but also as delayed medical consultation itself, which is associated with tangible clinical consequences.

Yousaf et al. in their cross‐sectional study from the United States, reported that 45% of acne patients received treatment recommendations from social media and only 31% of these recommendations were consistent with American Academy of Dermatology (AAD) guidelines [12]. Ünal et al. in their study encompassing 481 acne patients from Turkey, found that 78.3% of patients used social media for information about acne and did not share a substantial portion of this information with their physicians [13]. When these findings are evaluated collectively, it becomes apparent that patients first attempt to find solutions on their own using information obtained from social media, and in this process, through the use of dermatologically non‐indicated products, this pattern is associated with both barrier damage and more advanced clinical findings.

In our study, patients using TikTok as their information source demonstrated significantly higher rates of social media‐driven product purchasing (58.7% vs. 40.4%; *p* = 0.024), delayed medical consultation (66.7% vs. 49.0%; *p* = 0.028), and mean acne severity (2.31 vs. 2.03; *p* = 0.010) compared with Instagram users. This difference may be explained by the content structure and user demographics of the platforms. TikTok's short‐format, high‐engagement, and algorithm‐driven content delivery structure may direct users toward rapid, unreflective consumption behavior. In their multi‐platform content analysis, Thang et al. demonstrated that the educational quality of acne treatment‐related videos was lower on TikTok compared with other platforms and that content produced by non‐dermatologist sources received higher engagement [19].

Zheng et al. evaluated the content quality of the top 100 most‐liked acne‐related videos on TikTok using the DISCERN instrument and reported that the overall information quality contained serious shortcomings [20]. Irfan et al. confirmed in a similar analysis that DISCERN scores for acne content on TikTok were low across all categories [21]. Our study is among the first to reveal the patient‐level clinical correlates of these content quality findings: patients using a platform with a high concentration of low‐accuracy and low‐reliability content were associated with clinically more advanced disease findings, barrier damage, and complication manifestations.

Perhaps the most original finding of our study is that despite equivalent numbers of known terms (both 4.0), patients who followed only dermatologist accounts and those who followed only influencer accounts demonstrated dramatically different clinical outcomes. The barrier damage rate was 53.0% in the influencer‐following group versus 8.3% in the dermatologist‐following group, and severe acne rates were 42.6% and 12.2%, respectively. This finding suggests that the problem is related not to a lack of knowledge per se but rather to the source of information and the behavior it directs.

Logistic regression analysis supports this observation: in the model predicting social media‐driven product purchasing behavior, influencer following was identified as the strongest risk factor with OR = 5.68 (*p* < 0.001), while dermatologist following was a statistically significant protective factor with OR = 0.28 (*p* = 0.001). This striking contrast is consistent with findings reported by Bal et al. from Turkey, in which 97% of acne patients stated they would prefer the dermatologist when encountering conflicting information between a dermatologist and an influencer, yet 67% still sought information from social media influencers [22]. This paradoxical behavior suggests that despite patients' trust in dermatologists, the instant accessibility and algorithmic content delivery of content creators lacking medical qualifications have the potential to directly guide patient behaviors by bypassing rational decision‐making processes.

Another important finding of our study is the paradoxical relationship between dermocosmetic term awareness and clinical outcomes. Patients who knew more dermocosmetic terms paradoxically demonstrated higher rates of social media‐driven product purchasing (47.6% vs. 10.1%), barrier damage (35.9% vs. 16.2%), and delayed consultation (44.7% vs. 19.2%), contrary to expectations. This paradoxical finding raises important questions about the quality of knowledge acquired through social media.

Knowing terms such as retinol, salicylic acid, or AHA/BHA does not equate to possessing adequate knowledge about appropriate concentrations, frequency of use, potential interactions, and contraindications of these compounds. On social media, these terms are frequently presented alongside compelling marketing language and personal experience narratives, which may foster a perception of familiarity with these compounds that does not necessarily reflect accurate clinical understanding, an association compatible with—but not, on the basis of cross‐sectional data, proof of—increased social media‐driven product purchasing behavior. Indeed, our logistic regression analysis confirmed that the number of known terms is an independent predictor of social media‐driven product purchasing behavior (OR = 1.55; *p* < 0.001). This finding suggests that cosmetic knowledge lacking clinical context is associated with greater consumption behavior rather than informed decision‐making.

This paradoxical association is reminiscent of the knowledge–behavior gap described in the health literacy literature, although our cross‐sectional, non‐validated assessment does not allow a definitive claim regarding underlying cognitive mechanisms. Individuals with increasing levels of awareness are unable to translate this knowledge into correct clinical decision‐making processes and instead tend to experiment with more products. In the literature accessible to us, no study was identified that revealed this paradoxical relationship between cosmetic term awareness and clinical outcomes in acne patients; this finding represents one of the original contributions of our study to the literature.

The profile of patients assessed by the physician as cosmetic polypharmacy—concurrent use of multiple dermocosmetic products beyond the basic skincare routine recommended in current guidelines for acne patients (cleanser, active treatment agent, moisturizer, and sunscreen) without clinical indication—encapsulates the study's core findings [23]. In this group, barrier damage was present in 87.1%, social media‐driven product purchasing in 82.9%, and delayed consultation in 84.3%; the most frequently preferred information sources were TikTok (40.0%) and Instagram (35.7%). This profile concretizes the stepwise progression of social media‐driven cosmetic consumption behavior toward clinical consequences: a pattern in which patients exposed to content from creators lacking medical qualification show higher rates of social media‐driven product purchasing, concurrently use multiple products without clinical necessity, and exhibit barrier damage; in this group, delayed medical consultation co‐occurs with more advanced disease manifestations. Because of the cross‐sectional design, this stepwise description should be interpreted as a sequence of associations rather than a confirmed causal cascade. These findings have important implications for clinical practice. First, the social media usage habits and cosmetic product use histories of acne patients presenting to dermatology outpatient clinics should be routinely queried as part of the clinical history. Karadag et al. analyzed the current state of digital dermatologic communication in Turkey and emphasized that dermatologists should increase their evidence‐based content production on social media platforms [24]. Our data support this call with strong clinical evidence: it has been demonstrated that following dermatologist accounts reduces purchasing behavior and prevents barrier damage.

It should be noted that social media use is associated with later treatment seeking, particularly in the young patient population. The finding that barrier damage rate was as high as 75.0% among patients aged 12–17 years who purchased products through social media is concerning. In recent publications, social media‐driven skincare trends leading to irritant contact dermatitis and barrier damage in adolescents have been highlighted based on clinical observations, and this condition has been termed “digital dermatosis” [25]. Our findings support this warning with clinical data.

Increasing the visibility and accessibility of dermatology specialists on social media platforms is of importance. The protective effect of dermatologist following in our study (OR = 0.28 for barrier damage) and the low rate of severe acne (12.2%) among patients who followed only dermatologist accounts suggest that active participation of dermatology communities on social media may contribute to timely referral of patients to the treatment process and positive shaping of clinical outcomes. In this context, the development of verified health professional labeling systems and evidence‐based content guidelines by dermatology societies may be recommended.

### 4.1 Limitations of the Study

This study has several limitations. First, due to the cross‐sectional design, a causal relationship between social media use and clinical outcomes cannot be established; the associations identified are correlational in nature. However, the independent risk factors confirmed in logistic regression models and the dose–response relationship suggest the possible causal direction of the observed associations.

Second, the study is single‐center, limiting the generalizability of the results. Multicenter studies including patients from different regions and socioeconomic backgrounds may yield more comprehensive results. Third, the 7‐term structure used for dermocosmetic term awareness assessment does not constitute a validated scale. As no standardized cosmetic literacy scale specific to acne patients exists in the literature, this approach was developed pragmatically by the researchers. Development of a validated scale for this domain is recommended for future studies.

Fourth, barrier damage was assessed using standardized clinical criteria rather than objective biophysical methods such as transepidermal water loss (TEWL) measurement, corneometry, or skin pH‐metry. While clinical assessment by an experienced dermatologist using predefined criteria (≥ 2 of 3 findings: erythema, desquamation, and subjective symptoms of irritation) is a widely accepted approach in dermatologic practice and has been used in comparable cross‐sectional studies, the absence of instrumental measurements may have limited the sensitivity for detecting subclinical barrier dysfunction and reduces the reproducibility of our findings across different clinical settings. The use of a single trained observer mitigated inter‐observer variability but does not address the inherent limitations of clinical‐only assessment. Future studies are warranted to validate these associations using objective biophysical measurements of skin barrier function. Fifth, social media usage habits are based on patient self‐report, and recall bias is possible.

Sixth, several clinically relevant variables that may potentially confound the observed associations were not systematically evaluated in the present study. Socioeconomic status was only partially captured through education level; income, occupation, and geographic residence were not recorded, and the sensitivity analysis using education as a proxy did not reveal a significant independent effect. Psychiatric comorbidities such as depression and anxiety—which are highly prevalent among acne patients and may influence both health‐seeking behaviors and skincare‐related decisions—were not formally assessed. Constitutional skin characteristics such as Fitzpatrick skin type, atopic predisposition, and baseline sensitive skin status were not recorded, although these factors may modify susceptibility to irritant contact dermatitis. Prior acne treatment history, while available in our dataset and incorporated into the sensitivity analysis, was not analyzed at the level of specific therapeutic agents (e.g., topical retinoids, oral isotretinoin, hormonal therapy), which may differentially affect skin barrier function. Although the sensitivity analysis incorporating the proxy variables available in our dataset (prior treatment history and education level) demonstrated that the principal associations remained robust, future prospective and ideally multicenter studies should systematically incorporate these confounders—using validated psychometric scales, Fitzpatrick phototyping, and detailed treatment history—to further refine the observed relationships. Among the study's strengths are a large sample size (*n* = 408), a dual data collection structure incorporating both patient self‐report and physician assessment, identification of independent risk factors through multivariate analyzes, and the inclusion of patients not using social media as a natural control group.

## 5 Conclusion

This study demonstrates that social media‐driven cosmetic consumption is strongly and independently associated with skin barrier damage, delayed medical consultation, and increased disease severity in acne vulgaris patients. Social media‐driven product purchasing, influencer following, and the use of TikTok as a primary information source were associated with worse clinical outcomes, whereas following dermatologist accounts emerged as a protective factor.

The paradoxical increase in dermocosmetic term awareness was associated with greater product consumption and more advanced disease severity, barrier damage, and complication findings, suggesting that familiarity with dermocosmetic terminology acquired through social media is associated with risky consumption behavior rather than acting as a protective factor; this observation is hypothesis‐generating and warrants further investigation. These results emphasize the importance of increasing evidence‐based content production by dermatology specialists on social media and routinely assessing patients' digital health information sources in clinical practice. For future studies, the planning of multicenter, controlled, and longitudinal research evaluating the effects of dermatologist‐led digital content interventions on patients' cosmetic consumption behaviors and clinical outcomes is recommended.

## Author Contributions

**Orhan Şen:** conceptualization, methodology, data collection, formal analysis, writing – original draft, writing – review and editing. **Atıl Avcı:** conceptualization, methodology, supervision, writing – review and editing, critical revision.

## Funding

The authors have nothing to report.

## Ethics Statement

The authors confirm that the ethical policies of the journal, as noted on the journal's author guidelines page, have been adhered to and the appropriate ethical review committee approval has been received. Ethical approval for this study was obtained from the Kayseri City Hospital Non‐Interventional Clinical Research Ethics Committee (Decision No: 807, Date: 24.03.2026). The study was conducted in accordance with the principles of the Declaration of Helsinki and the Good Clinical Practice Guidelines. Written informed consent was obtained from all participants, and from the legal guardians of patients under 18 years of age.

## Conflicts of Interest

The authors declare no conflicts of interest.

## Data Availability

The data that support the findings of this study are available on request from the corresponding author. The data are not publicly available due to privacy or ethical restrictions.
